# Beyond TNBC: Repositioning of Clofazimine Against a Broad Range of Wnt-Dependent Cancers

**DOI:** 10.3389/fonc.2020.602817

**Published:** 2020-12-10

**Authors:** Jiabin Xu, Alexey Koval, Vladimir L. Katanaev

**Affiliations:** ^1^ Translational Research Center in Oncohaematology, Department of Cell Physiology and Metabolism, Faculty of Medicine, University of Geneva, Geneva, Switzerland; ^2^ Department of Biomedical Sciences, Faculty of Biology and Medicine, University of Lausanne, Lausanne, Switzerland; ^3^ School of Biomedicine, Far Eastern Federal University, Vladivostok, Russia; ^4^ Institute of Oceanography, Minjiang University, Fuzhou, China

**Keywords:** Wnt, repositioning, clofazimine, colorectal cancer, ovarian cancer, hepatocellular carcinoma, glioblastoma, breast cancer

## Abstract

Wnt signaling plays key roles in oncogenic transformation and progression in a number of cancer types, including tumors in the breast, colon, ovaries, liver, and other tissues. Despite this importance, no therapy targeting the Wnt pathway currently exists. We have previously shown that the anti-mycobacterium drug clofazimine is a specific inhibitor of Wnt signaling and cell proliferation in triple-negative breast cancer (TNBC). Here, we expand the applicability of clofazimine to a set of other Wnt-dependent cancers. Using a panel of cell lines from hepatocellular carcinoma, glioblastoma, as well as colorectal and ovarian cancer, we show that the efficacy of clofazimine against a given cancer type correlates with the basal levels of Wnt pathway activation and the ability of the drug to inhibit Wnt signaling in it, being further influenced by the cancer mutational spectrum. Our study establishes the basis for patient stratification in the future clinical trials of clofazimine and may ultimately contribute to the establishment of the Wnt pathway-targeted therapy against a diverse set of cancer types relying on the oncogenic Wnt signaling.

## Introduction

Translation of advances in technology and enhanced scientific knowledge of human diseases to novel therapeutics still faces high costs, long development time, and high attrition rate. Therefore, drug repositioning (also called repurposing) is considered an attractive strategy for identifying new uses of an approved or investigational drug that are outside its original medical indication (1, 2). Various data-driven and experimental approaches have suggested that drug repositioning strategy may offer certain advantages in terms of overall costs and time spent over developing a novel drug for a given indication (3).

Wnt signaling is a highly evolutionary conserved pathway playing major roles in the control of cell fate, proliferation and migration during organism development, but in healthy adult organs, it remains relatively silent (4). The aberrant activation of Wnt signaling is associated with tumorigenesis in many tissues (5). Efforts have been made in the past years for developing drugs against different Wnt pathway components for many cancer types, including breast cancer, colorectal cancer, ovarian cancer, leukemia, lung cancer, liver cancer, etc. Although some Wnt-targeting drugs have reached Phase II clinical trials, none has yet reached the market (5–7). Those being tested in clinical trials typically display strong side effects in the bone and intestine stemming from the on-target blunt inhibition of the Wnt pathway (6). These complications ask for the development of compounds with higher selectivity against cancer-specific Wnt pathways with less side effects (6).

Our previous works have provided comprehensive preclinical evidences that clofazimine, used in humans as an anti-leprosy agent with a well-established safety profile, is effective against triple-negative breast cancer (TNBC), an aggressive subtype of breast cancer (BC) currently lacking targeted therapy (8, 9). Clofazimine specifically inhibits canonical Wnt signaling in a panel of TNBC cells *in vitro*. In the *in vivo* mouse xenograft models of TNBC, clofazimine efficiently suppresses tumor growth through inhibition of the Wnt signal transduction. Clofazimine is also well compatible with doxorubicin, exerting additive effects on tumor growth suppression, producing no synergistic adverse effects. Together with the well-characterized pharmacokinetic profile and lack of serious adverse effects at therapeutically effective doses, this makes clofazimine a prime candidate for repositioning clinical trials (8, 9).

Colorectal cancer (CRC) is reported to account for 10.2% of all diagnosed cancers and 9.2% of deaths caused by cancer. It is considered as the second most common cancer worldwide (10). Multiple animal studies and data in human patients confirm that several mutations in the Wnt pathway are causative for CRC (11). Hepatocellular carcinoma (HCC) is the sixth most diagnosed cancer worldwide and the third leading cause of cancer-associated deaths (12). Mutations in key regulatory genes of the Wnt pathway promote growth, dedifferentiation and dissemination of HCC (13). Ovarian cancer (OC) is the fifth common cause of cancer-related deaths in women (14), and Wnt signaling plays an important role in tumorigenesis of a subset of ovarian cancers (15). Glioblastoma is the most common form of brain tumors, characterized by low responsiveness to treatment and poor prognosis (median survival <2 years) (16). Multiple oncogenic signaling pathways have been implicated in glioblastoma (17). Among them, Wnt signaling has been linked to the disease, especially in the context of cancer stem cells (18, 19).

This current work continues and extends the exploration of the potential of the Wnt inhibition and of the anti-proliferative properties of clofazimine for other Wnt-associated cancer cell types, including panels of CRC, HCC, OC, and glioblastoma cell lines. Upon treatment with clofazimine, we observed a correlation between cancer cell survival and the Wnt pathway levels; mutational spectrum of the cancer-associated genes is also found to influence the sensitivity to the drug. These data uncover the potential for expansion of the anti-cancer indications of clofazimine beyond TNBC and will be instructive for patient stratification.

## Material and Methods

### MTT Assay

50 μl of the indicated cell lines re-suspended at 60000 cells/ml for SW620, SW48, HepG2, Hep3B, PEO1, OVCAR3 (25,000/ml for HCT116, U87, U118, U251, or 30,000/ml for HT29, 35,000/ml for Huh7, 40,000/ml for LS174T, DLD1, SNU398, 50,000/ml for OVSAHO, KURAMOCHI) were added into each well of a transparent 384-well plate. The cells were maintained in DMEM containing 10% FBS for DLD1, SW620, SW48, LS174T, HT29, HCT116, Hep3B; RPMI without glucose containing 20% FBS and 10 µg/ml insulin for OVCAR3; RPMI without glucose containing 10% FBS, 2 mM sodium pyruvate, and 2 mM Glutamine for PEO1; RPMI without glucose containing 10% FBS, non-essential amino acids and 5 µg/ml insulin for KURAMOCHI; RPMI without glucose containing 10% FBS for OVSAHO; RPMI with 10% FBS for SNU398; DMEM without glucose containing 10% FBS and 1 g/L glucose for Huh7; DMEM without glucose containing 10% FBS, 1 g/L glucose and 25 mM HEPES for U87, U118, and U251; MEM without glucose containing 10% FBS for HepG2 and incubated at 37°C, 5% CO2 overnight. The medium of each well was replaced by 100 μl fresh medium the next day containing the indicated concentrations of clofazimine (Santa Cruz) or MSAB (Sigma-Aldrich). After incubation for 4–7 days (depending on the growth rate of line), the medium in each well was replaced by 50 μl of 0.5 mg/ml Thiazolyl blue (Carl Roth) solution in 1xPBS. The plates were incubated for 3 h at 37°C. Then the solution was removed and 50 μl DMSO was added into each well. Absorbance at 570 nm was measured in a Tecan Infinite M200 PRO plate reader.

### Luciferase Activity Measurement

The Wnt-induced and basal luciferase activity was analyzed as described (8, 20). Briefly, 50 μl of indicated cell lines at 60,000/ml for HCT116, U87, U118, U251, or 120,000/ml for KURAMOCHI, OVSAHO, or 84,000/ml for Huh7, or 96,000/ml for DLD1, SNU398, LS174T, or 144,000/ml for HepG2, Hep3B, SW620, OVCAR3, SW48, PEO1, BT20 were distributed in a white opaque 384-well plate. The cells were maintained in their respective culture mediums and incubated at 37°C, 5% CO2 overnight for attachment. Afterwards, the cells were transfected by the 1:1 mixture of the plasmid constitutively (under the CMV promoter) expressing Renilla luciferase (Addgene, Cambridge, MA, USA), and a reporter plasmid (Syngene). Transfection was carried out as described in the manufacturer’s protocol using 12 μg/ml of DNA and 40 μl/ml XtremeGENE 9 reagent (Roche). The next day, the medium of each well was replaced 30 μl fresh medium containing Wnt3a (500 ng/ml) (purified as described by (21) or GSK3β inhibitor (CHIR99021, Sigma-Aldrich) and clofazimine at 5, 10, and 15 µM concentrations (following 1h of pre-incubation with the compound), or MSAB at the indicated concentration. After overnight incubation, the supernatant in each well was removed and the luciferase activity was measured as described (21).

### Mutation Analysis

For comprehensive analysis of the relevant mutations in all the cell lines used, we have employed Broad Institute Cancer Cell Line Encyclopedia (CCLE). Since DLD1 and LS174T lines were absent in CCLE, we have used similar dataset generated in (22). The genes listed in **Table 1** in the respective lines were checked for mutations, copy number variations and fusion/translocation events. Since none of the latter two was found for any listed gene, only mutation data is shown in the tables. Top 10 mutated cancer-related genes (by OncoKB) for each type of cancer were selected using FireBrowse legacy datasets for TCGA available from CbioPortal (23). Clinical significance of the mutation was assessed with the COSMIC database (24) (see **Table 1** for details).

**Table 1:** Mutational spectrum of the 17 cancer lines used in the study.

Gene	Colorectal cancer cell lines							Hepatocellular carcinoma lines					Ovarian cancer cell lines					Glioblastoma cell lines				
	Frequency, %	HCT116	LS174T	HT29	DLD1	SW620	SW48	Frequency, %	SNU398(HBV^+^)^ℵ^	Huh7(HBV^-^)^ℵ^	Hep3B(HBV^+^)^ℵ^	HepG2(HBV^-^)^ℵ^	Frequency, %	OVSAHO	KURAMOCHI	OVCAR3	PEO1	Frequency, %	U118	U251	U87
APC	71.7			E1554fs/E853*	K993N/R727M	Q1338*	R2714C	3.2					2.2					0.3			
TP53	53.8			R273H	S241F	R273H/ P309S^§^		30.8	splice site	Y220C			87.7	R342*	D281Y	R248Q	G244D	29	R213Q	R273H	
KRAS	43	G13D	G12D		G13D	G12V		1.6					0.6					0.7			
LRP1B	17.9	A3541V				R4062*	W3255L	8.8					4.1					2.4		R4038K/C1109F	
FAT4	17.5	L1374P/ G2644fs					Y1215C/ D1743N/ V4509M	5.1					0.9					1.4			
FBXW7	17						G667fs	0.8					1.3					0.3			
PIK3CA	14.8	H1047R	H1047R	P449T	D549N/ E545K		G914R	3.5					0.6					11			
SMAD4	11.7			Q311*				1.1				V2906I	0		Y513C/C363S			0.3			
ATM	11.2	A1127V	S707P					2.4					1.3		M1644T/I2223M			1.4			
AMER1	11.2					A1012V		1.6		A229V/RA350fs			0.9					0.3			
CTNNB1	4.9	S45del	S45F				S33Y	26	S37C			25-140del^¥^	0.6					0.3			
ALB	1.3							11.5					0.9					1.4			
PCLO	8.5	Q3289*/A2819V/E1847D/K1219fs				T527I	K5063fs	9.4		C610S			1.9		P2485S/S956P	T115A		9.7			
ARID1A	9.4							8.6					0.9					0.7			
AXIN1	0.9				N307S			6.4			R146*		0					1			
ARID2	6.7							5.9					2.2					0.7			
KMT2D	5.8	V160M/P2442fs					R4904*/R755fs	5.6		splice site			0.3					1.7			
RB1	2.2							5.6			S576*		2.8					8.6			
NF1	3.6	P388T/T676fs						3.8					4.4	E1720D			2447_2448insL	11	K1444M	T676fs	LF1246fs
BRCA1	2.7		N810T					2.4					3.8					1.4			
BRCA2	4.5	IK2672fs					Q1782fs/A1689fs	2.9	R2973C				3.5		R2318*		Y1655*	1.4			
FAT1	5.8				L96I/A1480D/Q3251P/A3913V			2.4			I2857M		3.5					1			
LRRK2	8.5	C249Y						2.4					3.2		V1750L			1			
RNF213	6.3	R1601C					M1761V/E1173fs	2.9					2.8					1			
CDK12	4	P250H						0.8					2.8				E1311D	0.3			
PTEN	3.1							2.9					0.6					31	splice site	F241L	splice site
EGFR	4.5						G719S	1.6					2.2					26.6			
PIK3R1	4				P312L			1.1					0.3		splice site			11.4			
ATRX	2.2	T1529del			L629R	L359fs	ET886fs	1.9					0.6					5.9			N564S
KEL	3.6							0.5					0.9	R385S				5.2			
IDH1	1.3	S261L			G97D			2.1					0					5.2			

^¥^The in-frame deletion of amino acids 25–140 in CTNNB1 in HepG2 is not reported in the TCGA; this heterozygous deletion in HepG2 is however reported in a number of publications such as (27) and is confirmed to exist in our line of HepG2, see **Supplementary Figure 1**.

^§^TP51 in SW620 cells is homozygous mutant for two mutations, of which R273H is considered Tier 1, and P309S – Tier 4.

^ℵ^The Hepatitis B virus (HBV) status of the HCC lines was determined by analyzing the source pages of the line producers (ATCC and web.expasy.org/cellosaurus). Genes listed represent the 10 genes most frequently mutated in patients with each cancer type, given in the order of CRC, HCC, OC, and glioblastoma. Mutation frequency in patients is shown in “Frequency” column; if the gene is among the 10 most frequently mutated genes for the given cancer type, its mutation frequency value is highlighted in green. Underlined mutations are identified as homozygous by transcript expression ratio; del, complete amino acid deletion without frame shift; *, mutated to stop codon; fs, mutation causes frame shift. When several mutations are indicated for the same gene, they might be either on the same strand and on different ones. Color codes for the pathogenicity of mutations, as described in the COSMIC database (https://cancer.sanger.ac.uk/cosmic), with red representing Tier 1 mutations (pathogenic, clinical significance = 5 in Clinvar cancer-related diseases), orange representing Tier 2 (likely pathogenic, clinical significance = 4), yellow representing Tier 3, and non-color-coded representing all other mutations. For HCC lines, their Hepatitis B virus (HBV) status (positive or negative) is indicated.

### Western Blotting

Each cell line was seeded at 100,000 cells/well in 24 well plates. The next day, the medium was replaced with fresh medium with or without clofazimine at the indicated concentration pre-warmed at 37°C. After further 24 h incubation, the medium was removed, followed by washing with 500 μl of 1x PBS twice per well. The cells were lysed in the well by addition of 30 μl of ice-cold RIPA buffer [1x TBS, 4mM EDTA, 1% Triton, 0.1% SDS, 1x Protease inhibitor cocktail (Roche)] and incubated on ice for 10min. The cells were resuspended and then centrifuged at 18,000 g at 4°C to remove debris. 5 μl of the supernatants each were further analyzed by Western blot with antibodies against β-catenin (BD, catalogue No. 610154), α-Tubulin (Sigma, catalogue No. T6199-200), and c-Myc (Abcam, catalogue No. ab32072) at 1:1000 dilutions.

## Results

### Mutational Spectrum in a Panel of Cancer Lines Indicative of Overactivated Wnt Signaling

In our current investigation aiming at broadening the scope of repositioning of clofazimine against Wnt-dependent cancers, beyond TNBC previously analyzed by us (8, 9), we took six colorectal cancer (CRC) lines (SW48, HT29, HCT116, DLD1, SW620, and LS174T), four hepatocellular carcinoma (HCC) lines (SNU398, Hep3B, Huh7, and HepG2), four ovarian cancer (OC) lines (OVCAR3, OVSAHO, PEO1, and KURAMOCHI), and three glioblastoma lines (U87, U118, and U251). Analysis of the mutational burden in cancer-associated genes in individual patients has become a routine in clinics to choose better therapeutic strategy (25, 26). In an approximation to such patient-derived tumor analysis, we decided to first “stratify” our cancer cell lines based on their mutational profile, especially looking for mutations in genes encoding Wnt pathway components. As there is no standard list of mutations analyzed for all cancers, we employed a data-driven approach and listed 10 most frequently mutated cancer-related genes (by OncoKB) identified in the CRC, HCC, OC, and glioblastoma patient cohorts in the TCGA (see Methods for further details). The results of such analysis for our 17 cancer cell lines based on the Cancer Cell Line Encyclopedia are provided in **Table 1**.

As expected (11), a general mutational portrait of Wnt pathway activation is detected for CRC lines with the gain-of-function mutations in CTNNB1 (encoding β-catenin) noticeable in LS174T and SW48 cells (S45F and S33Y, respectively), along with loss-of-function mutations in APC in SW620 and HT29 lines (homozygous truncations in the protein). The remaining two lines also carry mutations in these two genes, although their pathological significance is not clear: HCT116 has a loss of S45 in β-catenin, and DLD1—two missense mutations in APC (**Table 1**).

Similarly, the mutational upregulation of the Wnt pathway is seen in three out of the four HCC lines, with pathogenic activating mutations in CTNNB1 [S37C in SNU398 and in-frame deletion of amino acids 25–140 in HepG2 (27)] or loss-of-function mutation in Axin1 in Hep3B cells (**Table 1** and **Supplementary Figure 1**).

In contrast, no genetic mutation-associated upregulation of the Wnt pathway can be observed in the OC and glioblastoma lines in our study (**Table 1**), agreeing with prior findings (17, 28) and suggesting that the Wnt pathway may utilize epigenetic, rather than genetic means of activation in these cancers, just as is the case in TNBC (29).

### Clofazimine Efficiently Suppresses Growth of the Panel of Colorectal, Hepatocellular, and Ovarian Cancer Cell Lines but Not Glioblastoma

**Figure 1:**
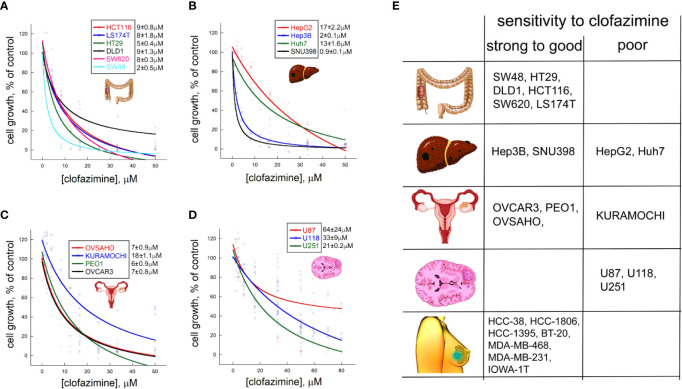
Different sensitivities to growth inhibitionl by clofazimine are revealed on a large panel of cancer lines from four distinct cancer types. Concentration-dependent decrease in cell proliferation in the MTT assay is seen for six colorectal cancer (CRC) lines **(A)**, four hepatocellular carcinoma (HCC) lines **(B)**, four ovarian cancer (OC) lines **(C)**, and three glioblastoma lines **(D)**. The curves are built on three to ten replicates for each tested clofazimine concentration. The calculated IC_50_ values are provided next to each cell line’s name as mean ± sem, n = 3 to 8). **(E)** Based on the IC_50_ values, and adding the data on seven TNBC lines previously published by us, a chart is created with the cell lines grouped into those having strong (IC_50_ ≤ 2 µM) to good (IC_50_ < 10 µM) sensitivity to clofazimine and those having poor (IC_50_ > 10 µM) sensitivity. A general picture emerges that TNBC, CRC, and subsets of HCC and OC, but not glioblastoma, lines has good to strong sensitivity to the drug.

In our previous work, we demonstrated that clofazimine has anticancer properties against six TNBC cell lines. MTT survival assay with clofazimine demonstrated an antiproliferative effect with IC_50_ around 3–8 µM (9). Concentration-dependent curves of the proliferation inhibition effects of clofazimine against six CRC cell lines (**Figure 1A**) show that the IC_50_ for CRC is from ca. 2 µM (SW48) to ca. 5 µM (HT29) and 8–9 µM for the remaining four lines (HCT116, DLD1, SW620, and LS174T), making CRC cells comparable to TNBC cells regarding their sensitivity to clofazimine.

In case of the four HCC cell lines we tested, clofazimine demonstrated a striking dichotomy: two lines, SNU398 and Hep3B, have low IC_50_ of 1–2 µM, whereas the other two—Huh7 and HepG2—are one order of magnitude less sensitive with IC_50_s of 13 and 17 µM, respectively (**Figure 1B**).

Of the four OC cell lines we tested (**Figure 1C**), three (OVCAR3, OVSAHO, and PEO1) behaved similarly, with the IC_50_ of clofazimine-mediated growth inhibition lying around 6–7 µM. In contrast, the KURAMOCHI cell line showed a considerably lower sensitivity, with IC_50_ of ca. 18 µM.

Finally, we tested three glioblastoma cell lines for their sensitivity to clofazimine. All the three lines (U87, U118, and U251) reveal poor sensitivity, with the IC_50_ of growth inhibition achieved only with 20–60 µM of clofazimine (**Figure 1D**).

This analysis of 6 CRC, 4 HCC, 4 OC, and 3 glioblastoma cell lines creates a matrix of growth inhibition sensitivities of cell lines of different cancer types to clofazimine. In an attempt to build a predictor of the sensitivity of any cancer cell line (or primary cancer cells) to clofazimine, we grouped these 17 cell lines into those displaying good (IC_50_ ≤ 10 µM) to strong sensitivity to clofazimine (IC_50_ ≤ 2 µM), on one hand, and those displaying poor sensitivity (IC_50_ > 10 µM). This cutoff was based on our prior extensive studies of the effect of clofazimine on TNBC growth *in vitro* and *in vivo* (8, 9), and together with these prior data on the high sensitivity of a panel of TNBC cell lines to clofazimine (9), this created the sensitivity table as shown on **Figure 1E**. In the following sections, we attempt to discern the reasons behind the high sensitivity of some cancer lines (and by inference – forms of cancer) to clofazimine, as compared to low sensitivity of others.

### Cancer Cell Lines Have Different Basal Levels of the Wnt Pathway Activity

As clofazimine has emerged as a Wnt pathway-inhibiting drug in the repositioning against TNBC (8, 9), we naturally assumed that the differential sensitivity of CRC, HCC, OC, and glioblastoma cell lines to clofazimine originates from different roles the Wnt pathway plays in the oncogenic transformation which led to these cancer cell lines. As the first step to evaluate the level of Wnt signaling in the lines, we measured the basal Wnt pathway activation in them, using the dual luciferase assay. In this assay, cells are double-transfected with the Wnt-specific TopFlash reporter plasmid and a Wnt-independent CMV-Renilla plasmid monitoring the general level of cell transcription. The Wnt pathway levels are then presented as relative luminescence units (RLU). The relative nature of this representation permits to compare Wnt pathway levels across cell types, being independent from individual cell transfection efficiency.

**Figure 2:**
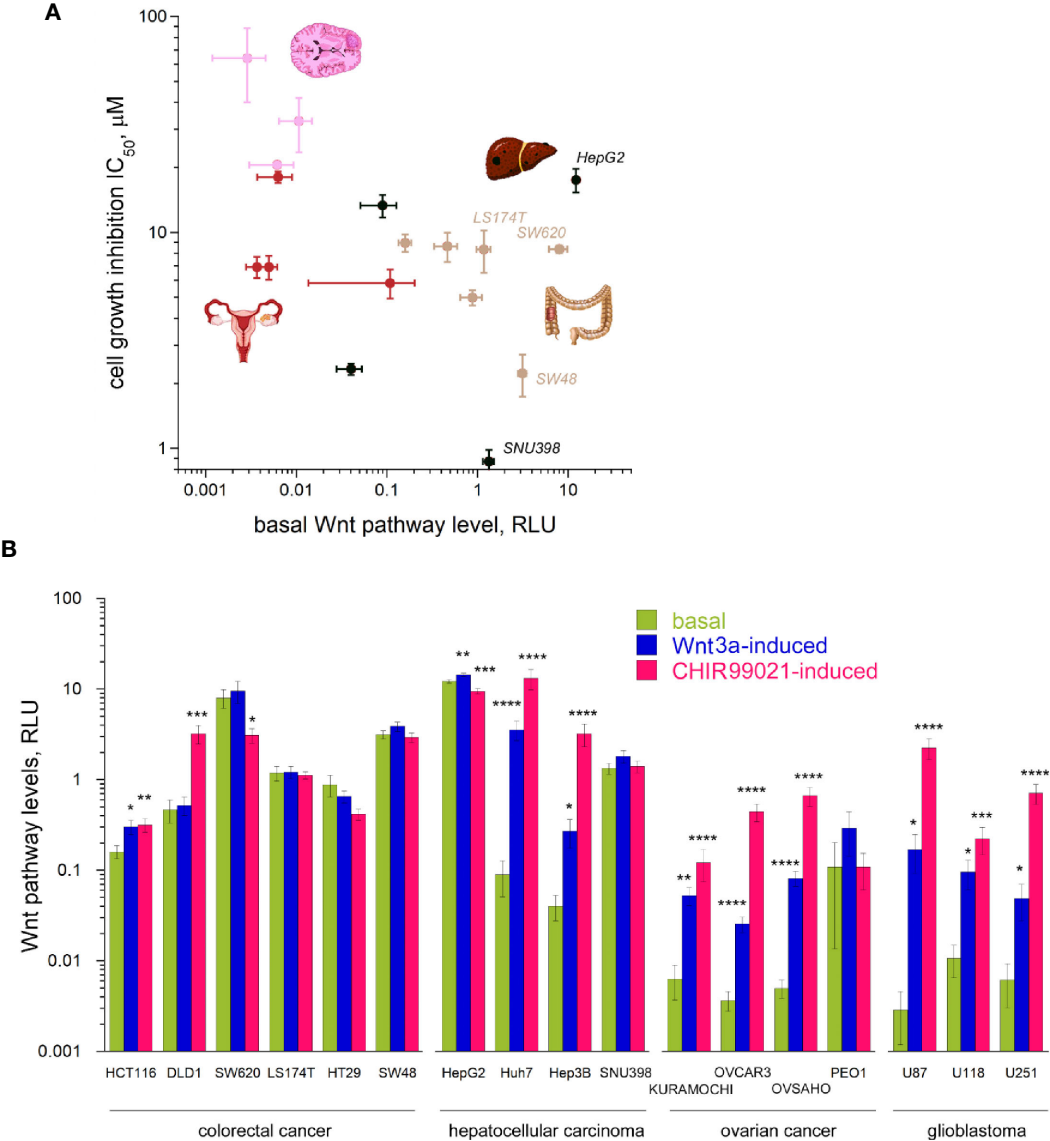
Wnt signaling pathway levels (basal and induced) across the 17 cancer lines. **(A)** The basal levels of Wnt signaling vary by 4–5 orders of magnitude across the cancer types and grossly correlate with the sensitivity to clofazimine. **(B)** Basal, Wnt3a-induced, and CHIR99021-induced levels of Wnt signaling across the cancer lines. Data are mean ± sem, n = 3 to 20. Statistical significance is determined by the t-test, p-value < 0.05 (*), <0.01 (**), <0.005 (***), or <0.001 (****). Note the log scales in **(A, B)**.

Using the dual luciferase assay, we found out that the basal levels of Wnt pathway activation in the cancer cell lines under our study differ from line to line dramatically, overall spanning across almost five orders of magnitude (**Figures 2A, B**). This vast range of the Wnt pathway levels agrees with the mutational spectrum of the cell lines we tested (**Table 1**, also see above). Indeed, while many cell types do not carry any mutations in the Wnt pathway components, like all the OC and glioblastoma cell lines studied in our work (**Table 1**), others do. Thus, the high basal levels in the Wnt pathway activation are linked with gain-of-function mutations in CTNNB1 or with loss-of-function mutations in APC or Axin1 (**Figures 2A, B**, **Table 1**, **Supplementary Figure 1**).

Apart from this huge span of the basal levels of the Wnt pathway in different cancer cells in our study, we observed a tendency of a correlation (R = 0.5245 after log transformation) between the basal Wnt level and the sensitivity of the cell line to clofazimine, so that the lower the basal level of Wnt pathway activation, the lower the observed sensitivity to clofazimine (**Figure 2A**). Next, we looked at the stimulated levels of the pathway in these cancer cell lines.

### Cancer Cell Lines Have Different Stimulated Levels of the Wnt Pathway

We then investigated how these 17 cell lines of four cancer types are capable to respond to activators of the Wnt pathway. We chose two means to achieve the activation. The first is a well-studied natural agonist of the pathway, Wnt3a, known to promiscuously stimulate multiple members of the FZD receptor family (21, 30). The second is CHIR99021—a small molecule inhibitor of GSK3β (31). While Wnt3a-induced signaling requires the complete Wnt transduction machinery to be active, all the way from the plasma membrane to cytoplasmic and to nuclear components, CHIR99021-induced activation bypasses the plasma membrane components of the pathway and can often lead to higher levels of the pathway activation (7). Using these two activators, we thus could probe the integrity of the Wnt transduction machinery across the cancer cell lines. This analysis uncovers the broad scope of Wnt transduction capacities in the four cancer types under study (**Figure 2B**). Certain cancer lines (SW620, HT29, SW48, LS174T, SNU398, HepG2), already having high levels of the basal Wnt activation through mutations in the Wnt pathway components, did not reveal a strong stimulation of the pathway neither by Wnt3a nor by CHIR99021 (actually a decrease in the Wnt signaling levels can be induced e.g. in HT29 or HepG2). DLD1 cells appear deficient in the pathway component(s) acting at the plasma membrane and did not respond to Wnt3a, yet were readily activated by the GSK3β inhibitor. And a third group (HCT116, Huh7, Hep3B, most OC and glioblastoma lines) showed robust pathway stimulation by both agonists.

While the stimulated levels of the pathway depict a reduced variation across the cell lines studied (by three orders of magnitude in case of stimulation with Wnt3a, and by two orders in case of stimulation with CHIR99021, as compared by the four-five orders of the basal level of the pathway activation), we do not see any correlation between these induced pathway levels and the sensitivity to clofazimine (data not shown).

### Clofazimine Inhibits Wnt Signaling in a Dose-Dependent Manner Across the Panel of Cancer Lines

**Figure 3:**
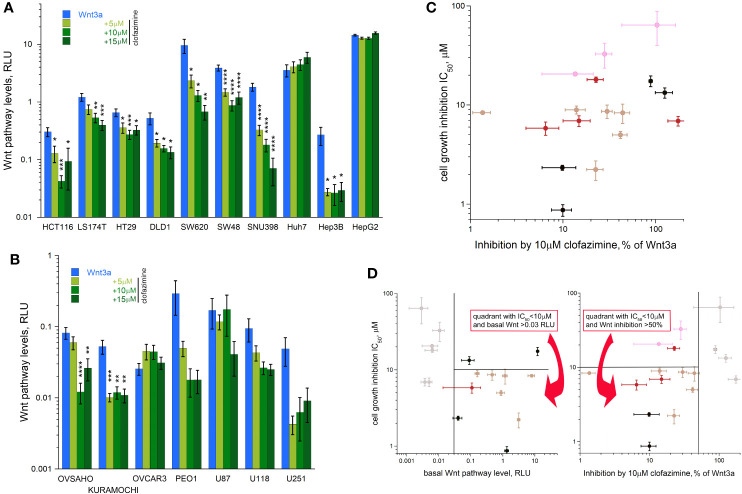
Clofazimine inhibits Wnt signaling across the cancer lines. Increasing concentrations (5-10-15 µM) of clofazimine are used to block Wnt signaling in the presence of Wnt3a in CRC and HCC lines **(A)** and OC and glioblastoma lines **(B)**. Note different log scales in **(A–C)** A gross correlation between the degree of Wnt pathway inhibition by clofazimine and growth inhibition by the drug can be notices across the cancer types. **(D)** A double criterion to predict sensitivity of a cell line to clofazimine can be built based on putting a threshold of a minimal level of basal Wnt pathway level (vertical line on the left panel) and the ability of 10 µM clofazimine to decrease pathway levels in the presence of Wnt3a by at least 50% (vertical line on the right panel). The cells falling inside the two quadrants as shown on the panels are those fulfilling the selection criteria and having the good to strong sensitivity to clofazimine (the horizontal line on the panels indicates the IC_50_ threshold of 10 µM). Note the log scale in **(C, D)**. Data presentation as in **Figure 2**.

We further analyzed effects of 3 concentrations of clofazimine (5, 10, 15 µM) on the Wnt3a-induced response in the TopFlash assay in the panels of CRC, HCC, OC, and glioblastoma cell lines (**Figures 3A, B**). As expected, clofazimine induced significant and specific inhibition of Wnt signaling in a dose-dependent manner in many of these lines. The following trends could be observed: i) inhibition of the Wnt signaling by clofazimine in cell lines having relatively low basal signaling levels and competent to respond to Wnt3a stimulation (with clofazimine thus bringing the signaling back to the basal levels): the Hep3B HCC line and most OC lines; ii) inhibition of the Wnt signaling by clofazimine in cell lines having high basal levels of the signaling and modestly or not at all responsive to extra Wnt3a stimulation (with clofazimine thus bringing the signaling well below the basal levels): all six CRC lines and the SNU398 HCC line; iii) no or no clear inhibition by clofazimine, regardless of the basal high (the HCC line HepG2) or basal low and Wnt3a-stimulated levels (the HCC Huh7 and glioblastoma lines) (**Figures 3A, B**). As an independent means to monitor the inhibition of Wnt signaling by clofazimine, we looked at the levels of the c-Myc protein—one of the well-established Wnt pathway-target genes (32) in representative cell lines. As can be seen in **Supplementary Figure 2**, in these cell lines, clofazimine, in the concentrations equivalent to its IC_50_ value in the cell proliferation assay (see **Figure 1**), robustly suppressed c-Myc levels.

A certain tendency of a correlation (R = 0.3697 after log transformation) could be seen between the extent of the Wnt pathway suppression by clofazimine and the sensitivity to the drug in terms of the cell growth inhibition, so that the stronger the ability of clofazimine to inhibit Wnt signaling, the more apparent is the growth sensitivity to it (**Figure 3C**). However, strong deviations from this tendency can be observed. The predictive power could be increased if the combination of the two analyses is applied: that of the basal Wnt signaling levels and that of the ability of clofazimine to suppress Wnt signaling (**Figure 3D**). In this case, thresholds could be imposed to filter out the cell lines having too low levels of basal Wnt signaling, and next removing the lines where clofazimine did not produce at least a 50% suppression of the pathway over that in the presence of Wnt3a (**Figure 3D**). In this manner, most of the cell lines fulfilling both Wnt signaling criteria showed good to strong sensitivity to the drug in terms of the growth inhibition. These cell lines are all the six studied CRC lines, two out of four studied HCC lines (SNU396 and Hep3B), and one or two OC lines out of the four studied. In contrast, all glioblastoma lines we have analyzed are filtered out.

In order to test if the clofazimine-sensitive cancer lines can be similarly affected by another Wnt pathway inhibitor, we used MSAB, methyl 3-{[(4-methylphenyl)sulfonyl]amino}benzoate. Interacting with β-catenin and promoting its degradation (33), MSAB acts at a downstream component of the Wnt pathway, as clofazimine also does (8, 9). Choosing the cancer cell lines showing responsiveness to clofazimine (**Figure 3D**), we found that MSAB efficiently suppressed both the Wnt signaling and cell proliferation in them (**Supplementary Figure 3**).

Thus, we conclude that subsets of cancer lines—and by inference, cancer cases—across different types of solid tumors reveal sensitivity to the Wnt pathway inhibition. The differences in the sensitivity to clofazimine we identify across the cancer panel can serve as the basis for future patient stratification in the precision oncology applications and are further considered in Discussion.

## Discussion

Clofazimine is a lichen-derived compound and was first synthesized and tested against tuberculosis (34), however, its efficacy against *M. tuberculosis* was considered insufficient. Instead, clofazimine demonstrated to be more potent against another mycobacterium, *M. leprae* (35). Thus, since early 1960s clofazimine was remarketed for the treatment of leprosy and is recommended by the World Health Organization as part of the standard multidrug therapy of leprosy (https://apps.who.int/iris/handle/10665/64636). Clofazimine’ efficacy in treating leprosy results not only from its antimicrobial activity, but also from the anti-inflammatory activity (36–38). Clofazimine is further reported to be useful for several infectious and non-infectious diseases, such as atypical mycobacterial infections, rhinoscleroma, pyoderma gangrenosum, necrobiosis lipoidica, severe acne, pustular psoriasis, and discoid lupus erythematosus (39). Beginning from the mid-90s, studies arise testing the anticancer potential of clofazimine, showing that it is effective against cultures of squamous hepatocellular carcinoma (40) and lung cancer (41) cell lines. However, only a few mechanistic studies on this effect have been done (9). Clofazimine was also tested for its anticancer activity against advanced hepatocellular carcinoma in patients, however, without a clear positive outcome (42, 43).

The most likely reason of the failure of clofazimine in the trials described above is the lack of proper stratification of the susceptible patients. Indeed, any targeted treatment requires a precise and data-driven knowledge of the target group of patients. Our current study is a significant step in filling this gap for clofazimine as an anti-Wnt cancer therapy, expanding the scope of clofazimine beyond TNBC to other Wnt-dependent cancers and in the same time narrowing down the clinical features of the tumors most susceptible to the compound. In our analysis, we chose multiple lines from four major cancer types: CRC, OC, HCC, and glioblastoma. Complemented with 6 TNBC cell lines from our previous studies (8, 9), this overall represents 23 cell lines from five cancer types tested for the sensitivity to clofazimine in terms of growth inhibition and Wnt signaling. This massive analysis shows that some cell lines—and by inference cancer types—are well sensitive to clofazimine, while others are relatively resistant to it. From our current and past studies, it can be concluded that all TNBC and CRC cell lines, subsets of HCC and OC lines, but not glioblastoma cell lines are sensitive to clofazimine and can be used in further experiments, as summarized in **Figure 1E**.

The grand conclusion of our work is that this differential sensitivity can be predicted by i) how high the basal level of the Wnt pathway in the cancer cell line is, and ii) how strongly clofazimine inhibits the Wnt signaling in these lines. Despite this general predictivity matrix, deviations still exist. We envision that these deviations can result from a) mutational and/or epigenetic portrait of each cancer line/case, and b) other mechanisms of action of clofazimine apart from its ability to block the Wnt pathway. We discuss these below.

Analysis of the mutational spectrum in the cancer lines we study (**Table 1**) provides some interesting clues into differences in the sensitivity to clofazimine found between cancer types but also within the same cancer type. Regarding the latter, it is intriguing to notice that although all six CRC lines we studied fall into the group of good to strong sensitivity to clofazimine (**Figure 1E**), the distinction between the four “good” lines (HCT116, DLD1, SW620, LS174T) and the two “strong” lines (SW48 and HT29) may lie in the KRAS mutation status. Indeed, SW48 and HT29 are the only two CRC lines of our group not bearing KRAS mutations. All the other four CRC lines contain gain-of-function mutations in this oncogene, which is expected to contribute to better cancer cell survival (44) and thus a right-shift of the curve of the clofazimine-mediated inhibition of cell proliferation (**Figure 1A**, **Table 1**). Regarding the former, the three glioblastoma lines in our study all harbor mutations in the top-mutated oncogene for this tumor: PTEN (**Table 1**). The poor sensitivity of these lines to the Wnt pathway inhibition by clofazimine might be explainable by the resultant overactivation of the oncogenic PI3K pathway (45), with the limited role played by the Wnt signaling in this cancer type or at least in the cell lines we had selected.

It is also intriguing to note that among the four HCC lines analyzed in our work, both clofazimine-sensitive ones (Hep3B and SNU398) originate from Hepatitis B virus (HBV)-positive HCC, while the two lines poorly sensitive to the drug (HepG2 and Huh7) are negative for HBV DNA (**Table 1**). HBV infection, in part through the viral protein HxB, is known to stimulate the Wnt pathway (46, 47), which may be the mode of Wnt pathway activation in HCC, which, as opposed to mutational activation (**Table 1**), is well sensitive to clofazimine inhibition. Potentially, this information may turn highly useful in future patient stratification in HCC clinical trials.

Another basis for the differential sensitivity of cancer lines to clofazimine, deviating from that to be expected based on the analysis of the Wnt pathway in these lines (**Figure 3D**), might be related to existence of other potential targets of clofazimine apart from the Wnt signaling in cancer cells. Indeed, clofazimine has been proposed to inhibit the mitochondrial Kv1.3 channels in leukemia (48) and pancreatic adenocarcinoma (49); other evoked mechanisms of action involve interference with PLA2 (40) or enzymes of the respiratory chain (41). The relative importance of the several clofazimine targets and mechanisms of action in a given cancer line can clearly contribute to the sensitivity of this line to the drug.

To summarize, we have established the broad sensitivity of cancer lines across different cancer types to clofazimine. This sensitivity grossly correlates with and can be predicted by the basal levels of Wnt signaling and by the ability of clofazimine to inhibit the Wnt pathway in the cancer lines. This work lays the ground for continued repositioning of clofazimine against a broad range of Wnt-dependent cancers and creates the basis for patient stratification in eventual clinical trials.

## Data Availability Statement

The original contributions presented in the study are included in the article/**Supplementary Material**. Further inquiries can be directed to the corresponding author.

## Author Contributions

JX performed experiments and analysis, and wrote the manuscript. AK performed and supervised experiments and analysis, and wrote the manuscript. VK led the project, supervised experiments and analysis, and wrote the manuscript. All authors contributed to the article and approved the submitted version.

## Funding

The work was funded by the Novartis Foundation for Medical-Biological Research grant #17C153 to VK. JX is a recipient of the Theodor et Gabriela Kummer fellowship.

## Conflict of Interest

The authors declare that the research was conducted in the absence of any commercial or financial relationships that could be construed as a potential conflict of interest.
