# Supplementary material for: Beyond TNBC: Repositioning of Clofazimine Against a Broad Range of Wnt-Dependent Cancers
Source: Front Oncol. 2020 Dec 10;10:602817. doi: 10.3389/fonc.2020.602817 (PMC7758533; doi:10.3389/fonc.2020.602817)
Supplement: Supplementary file 1 [file DataSheet_1.docx]

SUPPLEMENTARY MATERIALS include Supplementary Figures 1-3.

Supplementary Figure 1. β-catenin status of some HCC cells.

Mouse fibroblasts (L-cells) and human HCC lines Hep3B, SNU398 and HepG2 were tested for the presence and state of total β-catenin by Western blots. While L-cells do not have any basally active Wnt and resultantly no β-catenin (Koval et al., 2014), the three HCC lines have strong levels of β-catenin indicative of their high basal levels of Wnt pathway activation. Further, the HepG2 line shows two distinct bands of the protein: the wild-type and truncated; the electrophoretic mobility of the latter corresponds to the deletion of amino acids 25-140 described for this cell line (de La Coste et al., 1998). α-tubulin is stained on the same membrane for the loading control.


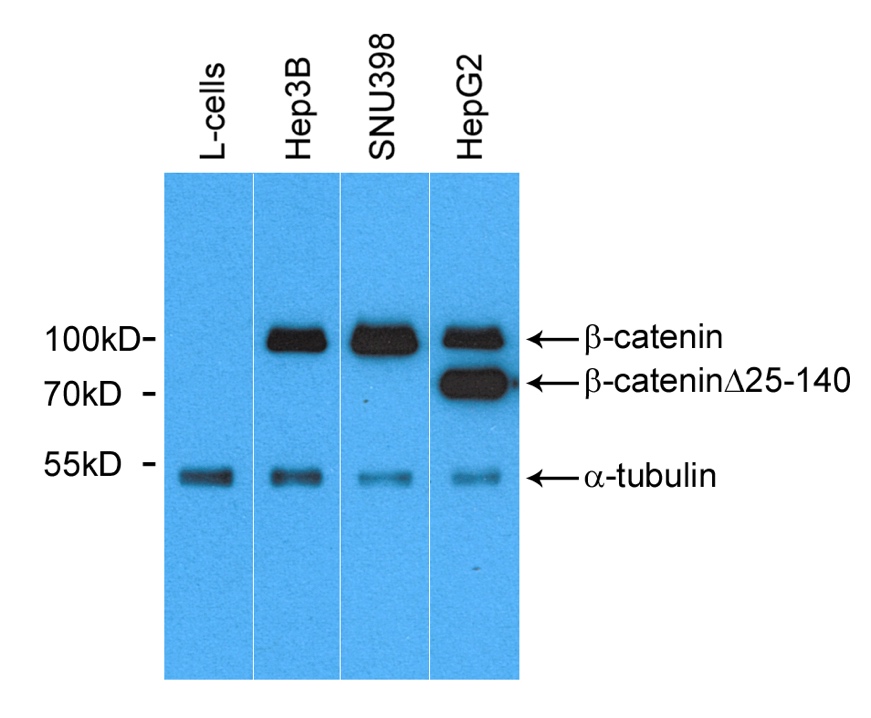


Supplementary Figure 2. Clofazimine decreases c-Myc levels in representative cancer cell lines.

Four CRC cell lines and one OC cell line were tested for the effect of clofazimine on the basal c-Myc protein levels, as measured by Western blots. Clofazimine concentrations corresponding to the IC_50_ levels in the cell proliferation inhibition assays (Fig. 1) were used. (A) Representative Western blots. (B) Quantification of three independent experiments, presented as the densitometry ratio of c-Myc and β-tubulin bands. Data is presented as mean ± sd. Statistical significance of the difference from control-treated cells is assessed by the Student’s t-test, p-value < 0.05 (*) or <0.01 (**).

Supplementary Figure 3. A Wnt pathway inhibitor MSAB efficiently suppresses Wnt signaling and cell proliferation in the clofazimine-sensitive cancer cell lines.

The six CRC cell lines, two OC, and two HCC cell lines were tested in the MTT assay (blue bars) and in the TopFlash assay in the presence of exogenous Wnt3a, as performed in Fig. 1 and Fig. 3, respectively. MSAB was used at the concentration of 20μM. All inhibitions are statistically different from the control conditions, p-value < 0.0005 by the Student t-test.


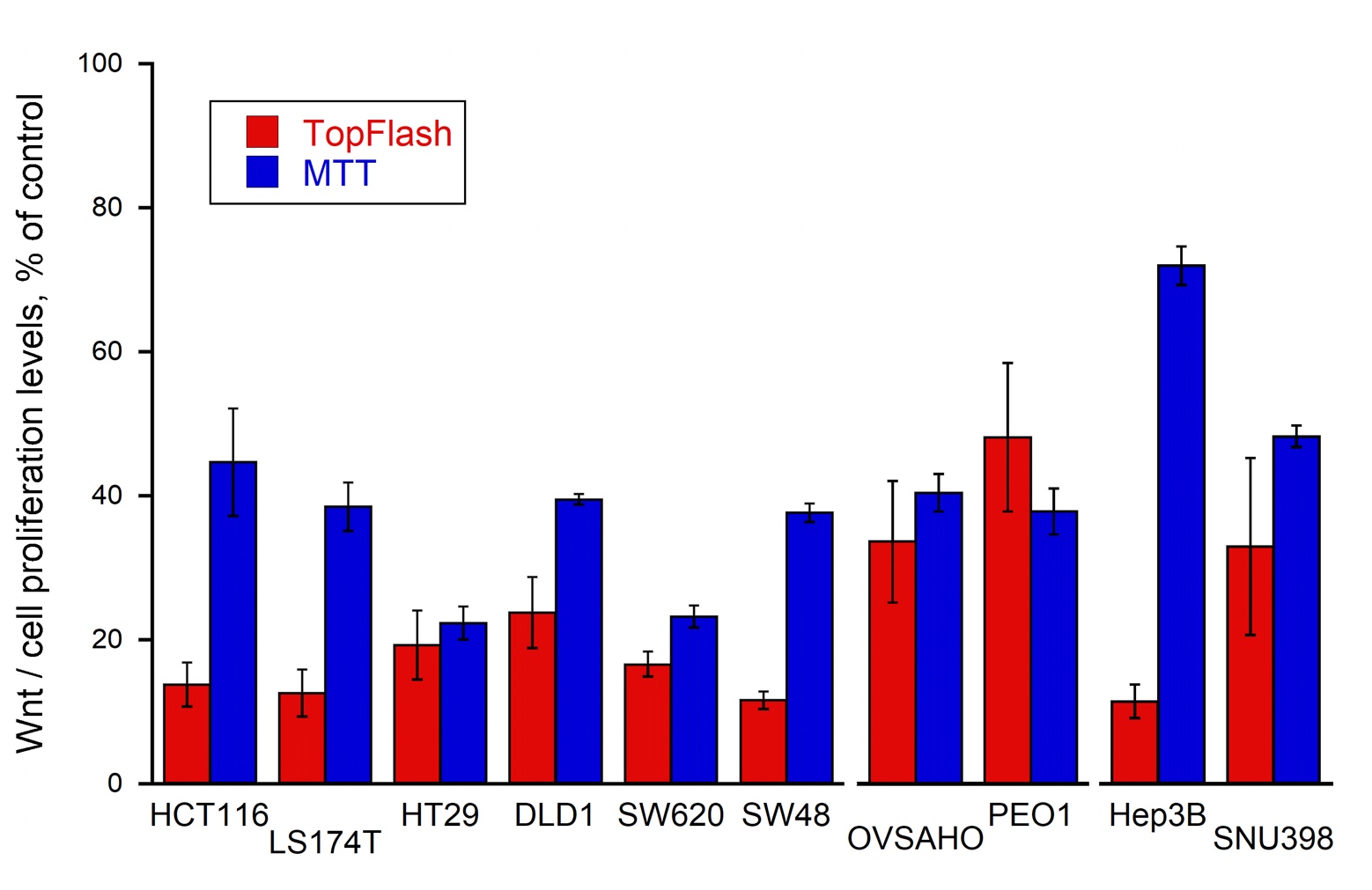


Supplementary references

De La Coste, A., Romagnolo, B., Billuart, P., Renard, C.A., Buendia, M.A., Soubrane, O., Fabre, M., Chelly, J., Beldjord, C., Kahn, A., and Perret, C. (1998). Somatic mutations of the beta-catenin gene are frequent in mouse and human hepatocellular carcinomas. *Proc Natl Acad Sci U S A* 95**,** 8847-8851.

Koval, A.V., Vlasov, P., Shichkova, P., Khunderyakova, S., Markov, Y., Panchenko, J., Volodina, A., Kondrashov, F.A., and Katanaev, V.L. (2014). Anti-leprosy drug clofazimine inhibits growth of triple-negative breast cancer cells via inhibition of canonical Wnt signaling. *Biochem Pharmacol* 87**,** 571-578.
